# Effects of Extended Underwater Sections on the Physiological and Biomechanical Parameters of Competitive Swimmers

**DOI:** 10.3389/fphys.2022.815766

**Published:** 2022-01-26

**Authors:** Santiago Veiga, Robin Pla, Xiao Qiu, David Boudet, Alexandre Guimard

**Affiliations:** ^1^Departamento de Deportes, Facultad de Ciencias de la Actividad Física y del Deporte—INEF, Universidad Politécnica de Madrid, Madrid, Spain; ^2^French Swimming Federation, Clichy, France; ^3^Institut de Recherche BioMédicale et d’Epidémiologie du Sport, IRMES, Paris, France; ^4^Institute of Sports and Sport Science, University of Kassel, Kassel, Germany; ^5^Club Natación Helios, Zaragoza, Spain; ^6^Université Sorbonne Paris Nord, Hypoxie et Poumon, H&P, INSERM, UMR 1272, Bobigny, France; ^7^Département STAPS, Université Sorbonne Paris Nord, Bobigny, France

**Keywords:** apnea, underwater undulatory swimming, swimming start, swimming turn, breath-holding, RPE, elite swimmers, dolphin kick

## Abstract

Despite changes in the underwater sections of swimming races affecting overall performance, there is no information about the effects of the apnea-induced changes on the physiological state of competitive swimmers. The aim of the present research was to examine the effect of changes in the underwater race sections on the physiological [blood lactate concentration, heart rate, and rating of perceived exertion (RPE)] and biomechanical (underwater time, distance, and velocity) parameters of competitive swimmers. Twelve youth competitive swimmers belonging to the national team (706 ± 28.9 FINA points) performed 2 × 75 m efforts under three different conditions, while maintaining a 200 m race pace: (1) free underwater sections, (2) kick number of condition 1 plus two kicks, and (3) maximum distance underwater. Overall performance was maintained, and underwater section durations increased from condition 1 to 3 as expected according to the experimental design. Heart rate and blood lactate concentration values did not show differences between conditions, but the RPE values were significantly greater (*F*_2, 36_ = 18.00, *p* = 0.001, *η*^2^: 0.50) for the constrained (conditions 2 and 3) vs. the free underwater condition. Underwater parameters were modified within the 75 m efforts (lap 1 to lap 3), but the magnitude of changes did not depend on the experimental condition (all lap × condition effects *p* > 0.05). Controlled increases of underwater sections in trained swimmers can led to optimizing performance in these race segments despite small increases of perceived discomfort.

## Introduction

The underwater segments of swimming races have reunited the interest of coaches and researchers in the last years, according to the increasing number of publications on the topic. These race segments represent the periods where swimmers stay underwater in apnea conditions after they dive or push off the starting or turning wall and before they emerge at the water’s surface for mid-pool swimming. According to FINA rules (FINA, 2021), the maximum distance that swimmers can travel underwater before breaking the water’s surface is 15 m from the wall.

There are several reasons that explain the increasing importance of the underwater race parts for overall swimming performance. When underwater, swimmers encounter much less hydrodynamic drag resistance (Marinho et al., 2009) and therefore achieve faster forward velocities than when surface swimming (Takeda et al., 2009). The loss of forward velocity in the different race laps seems to be less in the underwater vs. the surface-swimming segments (Veiga and Roig, 2016). In addition, the underwater swimming segments have a positive effect on the forward velocity of the subsequent surface swimming. Indeed, the first strokes after the starting or turning movements have been reported to be faster than mid-pool swimming, and this is believed to occur due to previous momentum from underwater swimming (Veiga and Roig, 2017).

In line with the aforementioned factors, analysis of the 100 m and 200 m races at the World Swimming Championships revealed that small changes in the underwater distance and velocity traveled by elite swimmers could have a great impact on the overall race results (Veiga et al., 2016). Indeed, the relative contribution of the underwater parts to the overall swimming race distances has increased considerably over the last 20 years (Veiga et al., 2014), and the percentage of non-swimming time (underwater) can even discriminate between swimmers of different skill levels (Pla et al., 2021). Accordingly, in recent years, procedures for swimming race analysis in elite competitions have incorporated a more comprehensive approach, where both times at fixed distances (i.e., 15 m start or turn times) and also individual underwater distances traveled by swimmers have been measured. In this way, race analysts can perform a more specific evaluation of the underwater race parts (Veiga et al., 2013b).

However, despite it being well-known that changes in underwater swimming segments can influence overall race velocity, the consequences of the apnea periods on the physiological state of swimmers during competitions are unknown. When underwater, swimmers have to deal with a well-known cardiovascular response that aims for a distribution of pulmonary and blood oxygen stocks preferentially to the heart and the brain (Lindholm and Lundgren, 2009) to limit the effect of a possible hypoxia (Alboni et al., 2011). This so-called diving response is characterized by bradycardia, peripheral vasoconstriction, and increased blood pressure, reduced cardiac output and blood flow, and increased sympathetic activity (Sterba and Lundgren, 1988; Foster and Sheel, 2005). Also, apnea leads to hypercapnia (Qvist et al., 1985), which has many consequences particularly on tissue oxygenation (Woorons et al., 2010), lactatemia (Graham et al., 1980), and probably discomfort (Woorons et al., 2007). The diving response (and the bradycardic phenomenon) is somehow in contradiction with exercise, which implies an increase in heart rate (Alboni et al., 2011), and this is why it is difficult to identify physiological patterns for apnea in the context of exercise performance and particularly in swimming. For example, in artistic swimming, Rodríguez-Zamora et al. (2012) described a rapidly developing tachycardia up to maximal levels during technical routines with interspersed periods of marked bradycardia in the exercise bouts performed in apnea.

There is a precedent in the swimming literature (Rozi et al., 2018) that compared the results of an anaerobic capacity test performed with two protocols, one with underwater start sections of 15 m for all participants and one with no underwater start sections. No differences were observed in swimming performance, lactate concentration, or heart rate. During surface swimming, Guimard et al. (2014, 2018) examined the effect of an apnea condition on short distances and observed a decrease in the arterial oxygen saturation and an increase in the RPE values of swimmers in their 400 m race pace. However, the physiological cost of the underwater race sections of competitive swimming that represent the longest apnea periods during races is still unclear. Therefore, the aim of the present research was to examine the effect of changes in the underwater race sections on the physiological and biomechanical parameters of competitive swimmers. It was hypothesized that despite providing an advantage in terms of average velocity, the extended underwater sections would lead to an alteration in the physiological parameters of competitive swimmers and a decrease in the performance of the last laps of simulated race distances.

## Materials and Methods

Twelve competitive swimmers (five males: 184.2 cm and 70.9 kg, and seven females: 170.1 cm and 58.9 kg) belonging to the national youth swimming team were recruited to participate in the present study. All of them competed for the national team in youth international competitions, and their personal-best times at their preferred stroke were, on average, 706 ± 28.9 FINA points. Their parents or legal tutors gave written consent for the experiment, and all procedures were designed according to the declaration of Helsinki and approved by the local university’s ethics committee.

The experiment was performed in a 25 m pool with a water temperature of 26°C and all test were conducted in the same training session. After a standardized warm-up consisting of 1,500 m with drills, some aerobic descending work and 25 m speed repeats, swimmers performed a testing set at their 200 m race pace designed to evaluate the role of underwater swimming on biomechanical and physiological parameters. The set consisted of two repeats of 75 m with a dive start at their preferred stroke (four swimmers at front crawl, four swimmers at backstroke, and four swimmers at butterfly) and with a one-and-a-half-minute rest between the first and the second repeat. The 200 m pace was calculated for each swimmer in relation to their personal best, and reference times for the 75 m repeats were given to swimmers before the commencement of the experiment. If variations greater than 1% in swimming velocity were observed in the first of the two 75 m repeats (compared to goal times), then some feedback was given to swimmers for the second repeat. Only data from the second repeat were collected. The set (2 × 75 m) was repeated three times in a fixed order with a minimum of 15 min of active rest between each set. Before the commencement of the second and third block, different indications were given to swimmers in relation to the underwater segments for each block. Details can be seen in Table 1.

**Table 1:** Experimental protocol of competitive swimmers to evaluate the role of the underwater swimming on biomechanical and physiological parameters.

Free underwater condition	Set 1	2 × 75 dive on 2′30	200 m pace (swimmers asked to count the underwater kicks per lap)
Constrained underwater conditions	Set 2	2 × 75 dive on 2′30	200 m pace with two additional underwater kicks in each underwater section after start and turns
	Set 3	2 × 75 dive on 2′30	200 m pace with the maximum distance covered in each underwater section after start and turns

The experimental protocol was part of a national team training camp, and dedicated staff guaranteed that swimmers maintained the same routine (sleep, diet, and lifestyle) for the 24 h preceding the test. Right after the completion of each set (around 2 s after the end of the set), swimmers’ heart rates were measured with a Garmin HRM Tri (Garmin Ltd., United States) monitor for a period of 30 s. The highest heart rate during that period was defined as the peak heart rate. To minimize potential instrumentation bias, this procedure was tested before the study protocol, and the swimmers were familiarized with this technique. Around 1 min after the completion of the set, capillary samples for blood lactate were collected from the ear lobe with Lactate Pro2 (Arkray Factory, Inc., Japan). According to the authors’ experience, for young swimmers performing a non-maximal effort of 75 m, 1 min post-exercise could be the most representative moment to estimate lactate peak. Around 30 s after the lactate measurements, for each swimmer, a rating of perceived exertion (RPE) was used to estimate the perception of effort with the Borg CR-10 category-ratio scale (Borg, 1982), which has been suggested as being efficient to estimate swimming intensity for swimmers (Psycharakis, 2011).

Two video cameras (Sony DCR-HC20E) recording at a frame rate of 50 Hz and situated on the public stands 5 m from the starting or turning wall (approximately 4 m above and 10 m away from the swimmers lane) were employed to record the experiment. Videos were analyzed with Kinovea software 0.8.27 (Copyright © 2006–2011, Joan Charmant & Contrib.), including the manual digitization of the swimmers’ head emersion from underwater by an experienced observer. Six control points represented by colored floating buoys surrounding the swimming-pool lanes were employed for calibration purposes, as previously done by Veiga et al. (2010). This allowed converting two-dimensional screen coordinates into real-space coordinates of the plane formed by the water’s surface. Two experienced coaches using a manual stopwatch Seiko SVAS003 (Seiko Watch Corporation, Japan) collected total time in seconds of each swimming repeat. The distance traveled underwater (m) in each of the three swimming laps was calculated from the starting or turning wall to the point of the swimmers’ head emersion, underwater time (s) was calculated from wall contact to head emersion, average underwater velocity (m/s) was obtained by dividing underwater distance and underwater time, and also the number of underwater undulatory kicks (*n*) was counted from the trials’ video footage. Finally, average surface swimming velocity (m/s) was computed from the total time and distance of the repeats minus the underwater segments.

The distribution of data was examined for normality using the Shapiro–Wilk test, and all parameters were graphed for visual inspection to screen for outliers. Two outliers were presented in the total underwater distance, and they were then excluded for statistical analyses. A repeated-measures ANOVA was performed according to the swimming condition (set 1, set 2, or set 3) and swimming lap (lap 1, lap 2, or lap 3). Bonferroni’s *post hoc* tests were used to verify localized differences. Data were expressed as mean ± SD, and the magnitude of differences (if they existed) was calculated by eta-squared effect sizes. All statistical analyses were conducted using R version 4.1.0. Significance was set at *p* < 0.05.

## Results

As designed in the experimental protocol, overall swimming times did not present a significant main effect between the three different conditions (*F*_2, 22_ = 0.86, *p* = 0.43, *η*^2^: 0.05), but this was observed in the total underwater distance, underwater time, and underwater velocity (*F*_2, 20_ = 19.55, *p* = 0.001, *η*^2^: 0.52 for distance, *F*_2, 22_ = 10.94, *p* = 0.001, *η*^2^: 0.50 for time, and *F*_2, 20_ = 4.25, *p* = 0.03, *η*^2^: 0.30 for velocity) as well as the surface swimming velocity (*F*_2, 20_ = 3.86, *p* = 0.04, *η*^2^: 0.28) traveled by swimmers. *Post hoc* test revealed longer distances and times underwater in the constrained (conditions 2 and 3) vs. the free underwater conditions, slower underwater velocity in the maximum underwater condition and slower surface velocity in condition 2 than the rest of conditions (Table 2). In particular, there was a main effect for the total number of underwater kicks (*F*_2, 22_ = 38.70, *p* = 0.001, *η*^2^: 0.68) with significant differences in each of the experimental conditions.

**Table 2:** Performance, underwater kicking, and physiological parameters of competitive swimmers in different underwater swimming conditions.

	Condition 1	Condition 2	Condition 3
Swimming time (s)	49.99 ± 5.77	49.83 ± 5.52	49.69 ± 5.58
Total number of kicks (*n*)	16.0 ± 8.1^b,c^	20.7 ± 8.9^a,c^	25.4 ± 11.1^a,b^
Total underwater distance (m)	26.13 ± 3.50^b,c^	30.94 ± 4.98	32.21 ± 6.40
Total underwater time (s)	14.23 ± 3.99^b,c^	16.74 ± 4.56	18.06 ± 5.47
Total underwater velocity (m/s)	1.86 ± 0.24	1.84 ± 0.24	1.76 ± 0.24^a,b^
Total surface swimming velocity (m/s)	1.45 ± 0.14	1.43 ± 0.15^a,c^	1.45 ± 0.14
Heart rate (bpm)	174.5 ± 10.3	176.1 ± 9.9	177.0 ± 10.7
Blood lactate (Mmol/L)	9.35 ± 2.55	8.96 ± 3.84	10.31 ± 3.97
RPE	7.61 ± 0.82^b,c^	8.29 ± 0.81	8.62 ± 0.81

^a,b,c^Statistically different from the first, second, or third underwater swimming conditions, respectively.

**Figure 1:**
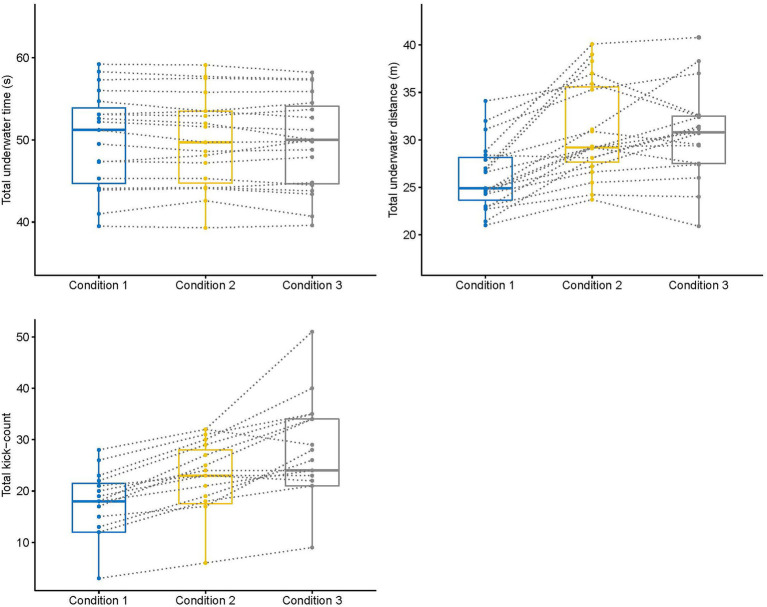
Change of underwater parameters of competitive swimmers from free (condition 1) to constrained (conditions 2 and 3) underwater swimming conditions. Box plots indicate minimum, first quartile, median, third quartile, and maximum of the data, respectively.

**Figure 2:**
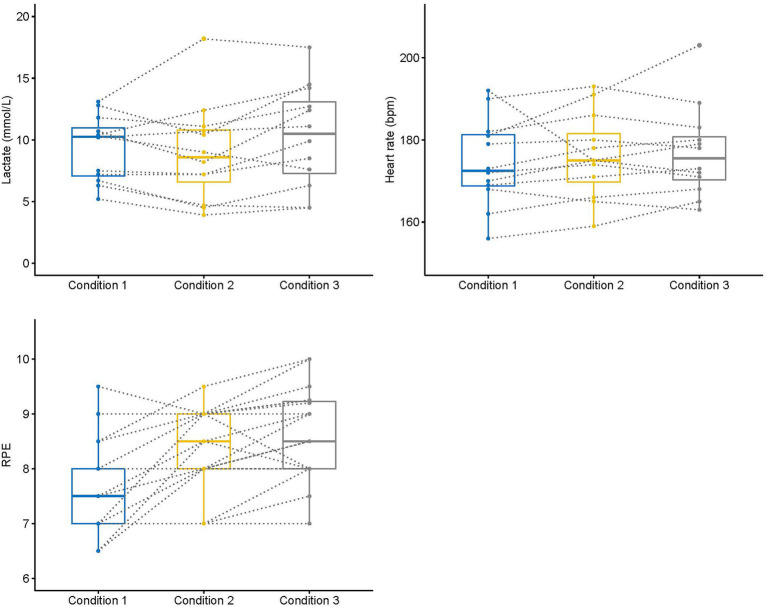
Change of physiological parameters and perceived exertion of competitive swimmers from free (condition 1) to constrained (conditions 2 and 3) underwater swimming conditions. Box plots indicate minimum, first quartile, median, third quartile, and maximum of the data, respectively.

In relation to the physiological measures, there were no main effects for the heart rate values (*F*_2, 22_ = 0.83, *p* = 0.44, *η*^2^: 0.07) or the blood lactate concentration (*F*_2, 22_ = 2.83, *p* = 0.08, *η*^2^: 0.20) of swimmers in the different experimental conditions, despite a tendency for blood lactate in condition 3 to be greater than in condition 2. However, there was a significant effect for the RPE (*F*_2, 22_ = 18.00, *p* = 0.001, *η*^2^: 0.50) with values in the constrained conditions being greater than the free underwater condition. Individual variation of physiological and biomechanical parameters is displayed in Figures 1, 2.

**Figure 3:**
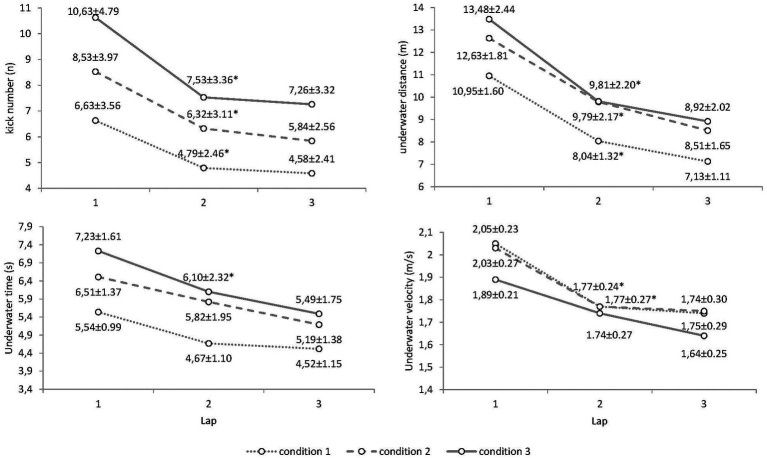
Evolution of the underwater parameters of competitive swimmers in different underwater swimming conditions.

Finally, the evolution of the biomechanical parameters on the 75 m repetitions presented a significant effect for the number of underwater kicks (*F*_2, 88_ = 55.35, *p* = 0.001, *η*^2^: 0.43) and also in the underwater distance (*F*_2, 88_ = 203.81, *p* = 0.001, *η*^2^: 0.74), underwater time (*F*_2, 80_ = 26.05, *p* = 0.001, *η*^2^: 0.32), underwater velocity (*F*_2, 80_ = 29.37, *p* = 0.001, *η*^2^: 0.42), and surface swimming velocity (*F*_2, 80_ = 74.36, *p* = 0.001, *η*^2^: 0.65) from lap 1 to lap 3. However, the magnitude of changes in the biomechanical parameters did not depend on the experimental condition (all lap × condition effects *p* > 0.05). Pairwise comparisons in the underwater segments between laps 1, 2, and 3 are presented in Figure 3.

## Discussion

The present research aimed to examine the effect of extended underwater race sections on the physiological and biomechanical parameters of competitive swimmers. Despite underwater sections of the start and turns being the fastest parts of swimming races and their relative contribution increasing at the elite level, there is no information in the literature about the effects of the apnea periods on the physiological state of competitive swimmers. Our results indicate an increased rate of perceived exertion when swimming for the same overall time with extended underwater swimming conditions but non-significant differences in the physiological parameters and similar modifications in the underwater segments from lap to lap. This is the first evidence in the literature for how changes in underwater swimming can affect competitive swimming performances.

As a first point of interest, the measurement of underwater sections in the different experimental conditions provided insightful data compared to previous research. When youth swimmers (male and females) spontaneously distributed their effort between underwater and surface sections (condition 1), they performed between one and two kicks less and traveled underwater distances around 1 m shorter than those reported for elite swimmers in the 200 m World Championships races (Veiga and Roig, 2016; Veiga et al., 2016). However, their average velocity during underwater swimming was considerably slower than that of male elite swimmers, who maintain values above 2 m/s (Pla et al., 2021). As expected, the average underwater velocity tended to decrease in the experimental conditions, where underwater sections lasted longer, but interestingly swimmers maintained their underwater velocity when they were constrained to adding only two underwater kicks per lap (condition 2). This could have strategic implications for swimmers and coaches, as athletes should aim to extend the underwater section provided the forward momentum does not fall too much (Veiga and Roig, 2017). When underwater distances were fully extended, while maintaining the total 75 m time (condition 3), the underwater distances of youth swimmers reached around 10 m with seven underwater kicks and altogether comprised 36% of the total swimming time. These values reached a similar contribution for the non-swimming times of international and national level male swimmers when at front-crawl events (Pla et al., 2021). Regardless of the experimental condition, distances traveled underwater from a dive start (lap 1) were 3–3.5 m longer than when pushing off the turning wall (laps 2 and 3). This represents a greater difference than the typical flight distances observed in elite youth swimmers during dive starts (Qiu et al., 2021), but differences were confirmed by the greater number of underwater kicks observed in lap 1 vs. laps 2 and 3 (Figure 3).

The evolution of underwater parameters displayed a typical decrease in distances and time spent underwater from lap to lap (Figure 3), probably explained by a continuous rise in blood lactate concentration during the race coupled with an increased over-time hypoxemic stimulus that probably increased the perception of effort (RPE). The time of the apnea and the exercise preceding the underwater swimming was also longer; however, when following a turn (laps 2 and 3) than during the dive start (lap 1), which could have a cumulative effect on the 75 m efforts. Youth swimmers in the present research were able to maintain the kick number and the underwater distances in the last turn in all the experimental conditions, as previously observed in elite swimmers (Veiga and Roig, 2016). In addition, underwater velocity values displayed practically no decrease in the last lap (Figure 3). This is something previously observed in elite races that confirms the importance of underwater sections to compensate for the loss of velocity during the surface swimming laps (Veiga and Roig, 2016). However, an important finding of the present research was that the changes of underwater sections did not depend on the apnea condition. Despite extended underwater sections revealing an increase in the rate of perceived exertion (Table 2), swimmers were able to maintain similar underwater changes from lap to lap when extending the underwater sections. Previous research on pacing has reported a lower performance level at the end of races when athletes have expended too much energy in the first part (Rodriguez and Veiga, 2018). However, our results indicate that when focusing on the underwater kick number, swimmers were able to improve their underwater performance despite an unavoidable increase in discomfort. Maybe the switching of attention from an internal focus (the difficulty and discomfort of apnea) toward an external focus (kick number) could represent a useful resource for improving performance (Lohse and Sherwood, 2011; Becker and Fairbrother, 2019) in swimming. Of course, simulations of race conditions in the present research occurred in 75 m efforts, where the number of consecutive turns was lower than in real competitions. However, this is the first study in the literature that examines underwater sections in 25 m pool races except that by Veiga et al. (2013a), which examined the 200 m backstroke event only.

For the heart rate values, no significant differences were observed between the experimental conditions. It is well-known that the magnitude of the diving response (and the consequent bradycardia) does not depend on the depth of diving but rather the length of the apnea (Schagatay and Andersson, 1998; Schagatay et al., 2000; Bosco et al., 2008). Therefore, it could be assumed that longer underwater swimming sections may imply a decrease in heart rate because of a more pronounced immersion time. This may be true for the present study, but the fact that heart rate was collected at the end of the 75 m efforts (and not at the end of the underwater sections) may hinder this conclusion. It could happen that bradycardia occurring during extended underwater sections may be counterbalanced by a bigger increase in heart rate after the underwater part, leading to similar heart rate values at the end of the effort. Only if checking the instantaneous heart rate during efforts, as previous studies have done for artistic swimming (Rodríguez-Zamora et al., 2012) and surface swimming (Guimard et al., 2014, 2017, 2018), could it be possible to draw solid conclusions about this. Also, another factor that could hinder conclusions is the large individual variability observed in the bradycardia response to apnea, as found in the study by Lindholm et al. (2002). The decrease in heart rate can vary from 15 to 40%, but a small proportion of healthy individuals can develop bradycardia up to 20 beats per minute (Alboni et al., 2011). It is interesting to note that previous studies on swimming have shown that the expected bradycardia was only observed in athletes unable to maintain their performance in apnea conditions (referred to as “with a bad apnea capacity”) during either 4 × 25 m (Guimard et al., 2014) or 50 m with fins (Guimard et al., 2017). Considering the high level of the swimmers, the present study was probably carried out by athletes “with a good apnea capacity” (Guimard et al., 2017), capable of maintaining an optimal cardiac output under conditions of intense dynamic apnea and thus their performance. Indeed, this could be confirmed by the similar relative decrease in underwater parameters lap to lap regardless of the longer apnea times requested (Figure 3).

Since some studies have found an increase in lactatemia in dynamic apnea (Matheson and McKenzie, 1988; Kume et al., 2013, 2016), one might logically expect that blood lactate concentration would be increased under conditions, where the duration of apnea is longer. However, there is some controversy in the literature as some swimming studies and other exercise modalities have never found an increase in lactatemia in apnea (Kjeld et al., 2009; Guimard et al., 2014, 2017, 2018). The present study presented no statistical differences between conditions but an increasing tendency of blood lactate concentration when underwater distances were maximum (condition 3). This could be explained by the swimmers’ effort regulation between the underwater and surface segments of 75 m laps. Indeed, swimmers had to keep the same overall time in the 75 m efforts, while modifying the extension of the underwater sections. Therefore, they could have adjusted the underwater over the surface swimming to complete the trial in the required pace. Results indicate that, in condition 2, swimmers maintained their underwater velocities but they slowed down on the surface swimming compared to the free underwater (condition 1). On the other hand, in condition 3, swimmers decreased their underwater velocities but maintained the swimming pace on the surface compared to condition 1 (Table 2). We can hypothesize that if swimmers had to swim the 75 m distances at a maximal effort with constrained underwater swimming, they probably would not be able to sustain this effort and maybe some differences in lactate concentration could be observed. In addition, the fact that lactatemia in the blood reflects the production but also the use of lactate as an energy substrate in apnea (Joulia et al., 2002) could hinder some differences in the lactatemia between conditions. Another suggestion is that in apnea the hypercapnia, i.e., the accumulation of CO_2_ (and therefore H^+^), could limit the output of muscle lactate (accompanied by H^+^; Lancha et al., 2015). Nevertheless, in the present study, only one blood lactate sample was taken for each swimmer at 1 min post-exercise and this may be too early. The procedure was employed to avoid interruptions on the training session and also based on the authors’ experience with young swimmers performing a non-maximal effort, where 1 min post-exercise samples were considered the most representative to estimate lactate peak. However, the phenomenon of vasoconstriction caused by the diving response could induce a delayed release of muscle lactates into the blood stream (Guimard et al., 2014). In a further study, it would be interesting to collect lactatemia at different times after the end of the race in order to identify the kinetics of changes and the peak of lactatemia.

Despite the lack of changes in the physiological markers, competitive swimmers reported a higher RPE in the constrained (conditions 2 and 3) vs. the free underwater swimming condition, and this would reveal a higher intensity perceived by the swimmers when they performed longer underwater sections. A previous study had reported higher RPE in swimmers in apnea than in normal breathing conditions when swimming at a 400 m race velocity (Guimard et al., 2018). The reason for this is the apnea-induced hypercapnia associated with prolonged exercise duration (Woorons et al., 2007), which causes discomfort, and it could also be the reason for swimmers perceiving a higher level of effort during longer underwater swimming. Lack of differences in RPE between the conditions 2 and 3 (where swimmers performed maximum underwater distances) could be explained by the lack of differences in the total underwater distances between conditions (Table 2), despite the tendency for swimmers to perform one more kick in condition 3. Swimmers tried to maximally extend the underwater swimming in condition 3, but their underwater kicking was probably not efficient enough to maintain the distance per kick at the end of underwater sections (Zamparo et al., 2012). Therefore, swimmers should probably try to extend underwater swimming provided: (1) the underwater kicking efficiency is keeping at the same level, (2) the average underwater velocity does not slow down, (3) the evolution from lap to lap is maintained, and (4) the physiological parameters and RPE do not present large modifications. In this context, the use of an RPE-type scale seems to constitute an interesting tool to manage the internal load related to the different apnea conditions while swimming.

Results of the present research involve some limitations that should be acknowledged in order to adequately draw conclusions. First, 75 m distances were not swum at a maximal effort, which may allow swimmers to regulate their effort between the underwater and surface swimming segments. Future studies where maximal efforts are performed by competitive swimmers could inform about the overall effect of changes in underwater parameters. Second, the data collection about the physiological parameters was after the end of the event and not after the end of the underwater part. This could explain why few differences in lactatemia or heart rate were observed between conditions considering that the final time was the same for each condition. Also, a measurement of gas exchange and blood samples right after each underwater section could inform in particular about the kinetics of O_2_ and CO_2_ to further clarify the effects of the experiment. Third, the fixed order on the experimental conditions could have some influence on reported data. This experimental design allowed to examine the spontaneous underwater pattern of swimmers during set 1, but it could have also affected reported values of RPE after the first set. Finally, data collection was performed during 75 m efforts at the swimmers’ preferred stroke that did not represent an official competitive distance, although it allowed for the research purposes to repeat different experimental conditions within the same experiment.

## Conclusion

The 200 m race pace efforts of competitive swimmers displayed an increased rate of perceived exertion when increasing the underwater swimming duration but non-significant differences in the physiological parameters. In addition, the evolution of the underwater parameters from lap to lap displayed similar changes in free vs. extended underwater swimming conditions. Effort regulation between the underwater and surface sections could explain the lack of differences in the physiological parameters of swimmers despite a higher effort perceived during longer underwater swimming. The control of the underwater kick number was revealed as a useful resource to optimize the underwater swimming performances provided the average underwater velocity was maintained. Also, the use of an RPE-type scale could represent an interesting tool to manage the internal load in different apnea conditions while swimming. However, in order to fully understand the changes in underwater sections on performance, testing on maximal swimming efforts should be conducted.

## Data Availability Statement

The raw data supporting the conclusions of this article will be made available by the authors, without undue reservation.

## Ethics Statement

The studies involving human participants were reviewed and approved by Comité Etico Universidad Politécnica de Madrid. Written informed consent to participate in this study was provided by the participants’ legal guardian/next of kin.

## Author Contributions

SV and DB contributed to the conception and design of the study. DB and XQ organized the database. XQ performed the statistical analysis. SV, XQ, RP, and AG performed the data analysis and the interpretation of the data. SV, RP, and AG contributed to the preparation and critically revised the manuscript. All authors contributed to the article and approved the submitted version.

## Conflict of Interest

The authors declare that the research was conducted in the absence of any commercial or financial relationships that could be construed as a potential conflict of interest.

## Publisher’s Note

All claims expressed in this article are solely those of the authors and do not necessarily represent those of their affiliated organizations, or those of the publisher, the editors and the reviewers. Any product that may be evaluated in this article, or claim that may be made by its manufacturer, is not guaranteed or endorsed by the publisher.
